# CTOS Board of Directors 2001–2002

**DOI:** 10.1080/135771402100059546

**Published:** 2002-09


					CTOS BOARD OF DIRECTORS 2001-2002
Lee Helman, M.D., President
Mark Gebhardt, M.D.,Vice President
Robert S. Benjamin, M.D., Secretary
Ole Steen Nielsen, M.D.,Treasurer
Jaap Verweij, M.D., Past President
Franco Gherlinzoni, M.D., (1999-02)
Michael Simon, M.D., (1999-02)
Scott Nelson, M.D., (1999-02)
Martine van Glabbeke, M.D., (1999-02)
Ephraim Casper, M.D., (2000-03)
Andrew Huvos, M.D., (2000-03)
Martin Robinson, M.D., (2000-03)
Junya Toguchida, M.D., (2000-03)
Kristy Weber, M.D., (2001-04)
JeanYves Blay, M.D., (2001-04)
Thomas DeLaney, M.D., (2001-04)
Pancras Hogendoorn, M.D., (2001-04)
H.J. Hoekstra, M.D., (2001-04)
Sarcoma (2002) 6, S57
ISSN 1357-714X print/ISSN 1369-1643 online/02/0200S57-00 (c) 2002 Taylor & Francis Ltd
DOI: 10.1080/135771402100059546
CTOS abstracts S59
Thursday, October 31
1:00 pm - 6:00 pm Registration, Poster Set- Up
6:00 pm - 7:00 pm Welcome Reception
Friday, November 1
7:00 am Registration, Continental Breakfast
7:00 am Executive Committee Meeting
8:00 am Welcome, Opening Announcements: Lee Helman, CTOS President & George Demetri,
Program Chair
8:15 am HOW WE IMAGE SARCOMAS AND APPROACH THE MANAGEMENT OF
PATIENTS WITH SARCOMAS
8:15 am #39 The Role Of Ultrasonography To Evaluate Chest Wall Sarcomas
Antonio Briccoli, Italy
8:30 am #78 High-resolution Intravascular Ultrasonography To Assess Vessel-
invasiveness Of Soft Tissue Sarcoma
Peter Hohenberger, Germany
8:45 am #27 Predictive Value Of Gadolinium Enhancement In Differentiating
Atypical Lipomas (well-differentiated Liposarcomas) From Benign Fatty
Tumors
Matthew J. Panzarella, USA
9:00 am #61 Fdg- Positron EmissionTomography (pet) In Staging And Managing
Patients With Soft Tissue Sarcomas
Thomas F. DeLaney, USA
9:15 am #31 Fluorodeoxyglucose Uptake In Adult Soft Tissue Sarcoma Predicts
Risk Of Recurrence After Chemotherapy
Scott M Schuetze, USA
9:30 am #48 Should Soft Tissue Sarcomas Be Treated At A Specialist Centre?
Robert Grimer, UK
9:45 am #55 Positive Margins After Attempted Wide Excision Of A Soft Tissue
Sarcoma: Is Further Excision Necessary?
Edward Y. Cheng, USA
10:00 am Discussion
10:15 am Coffee Break, Poster Viewing
10:45 am NEW DIAGNOSTIC TOOLS - cDNA MICROARRAY DATA AND
INTERPRETATION
10:45 am #73 Microarray Analysis Of Malignant Fibrous Histiocytoma
Jay S Wunder, Canada
11:00 am #49 Genome-wide Analysis Of Gene Expression In Synovial Sarcoma
Using A Cdna Microarray
Junya Toguchida, Japan
11:15 am #24 Differential Gene Expression In Leiomyosarcoma.
Keith M Skubitz, USA
11:30 am #35 Antisense Inhibition Of Hyaluronan Synthase-2 In Human
Osteosarcoma Cell Line, Mg-63, Inhibits Hyaluronan Retention And
Tumorigenicity Of The Cells
Yoshihiro Nishida, Japan
11:45 am #22 Neoplastic Transformation Of Primary Human Osteoblasts Over-
expressing The Met Receptor By Means Of TransductionWith Lentiviral
Vectors
Riccardo Ferracini, Italy
2002 CTOS Meeting Program
S60 CTOS abstracts
12:00 pm #71 (Young Investigator Award Winner) Targeting Met Oncogene In
Human Osteosarcoma By K-252a Tyrosine-kinase Inhibitor: A New
Therapeutic Approach
So a Avnet, Italy
12:15 pm Discussion
12:30 pm - 1:45 pm Lunch, Poster Viewing
12:30 pm - 1:45 pm Board of Directors Meeting
1:45 pm NEW LABORATORY AND CLINICAL TRIAL DATA
Amifostine and Ifosfamide
1:45 pm #46 Differential Effects Of The Radioprotectant Amifostine On Ewing's
Sarcoma And Human Monocytes In Culture
Matthew J. Allen, USA
2:00 pm #37 A RandomizedTrial Of AmifostineWith High-dose AlkylatorTherapy
In Ewing Sarcoma: A Children's Oncology Group Trial (p9457)
Mark L Bernstein, Canada
2:15 pm #20 Randomised Phase 3 Trial Of Two Investigational Schedules Of
IfosfamideVersus Standard Dose Doxorubicin In Patients With Advanced
Or Metastatic Soft Tissue Sarcoma (asts).
Paul Lorigan, UK
Cartilagenous Tumors
2:30 pm #17 Expression Of The Ihh/pthrp And Bmp Receptors Has Diagnostic
Relevance In Cartilagineous Lesions
Luca Sangiorgi, Italy
2:45 pm #19 Dedifferentiated Chondrosarcoma: Updated OutcomesWith Current
Treatment Approaches As Compared To Those Prior To 1984
Ian Douglas Dickey, USA
Desmoid Tumors
3:00 pm #50 Desmoid Tumors. A Clinical Review Of 30 Patients With MoreThan
20Years Follow-up
Mikael Dalen, Sweden
3:15 pm #30 Unexpected Severe Toxicity Of Low Dose Metothrexate And
Vinblastine In Patients With Desmoid Tumours
Bert van Geel,The Netherlands
Radiotherapy Dosing and Delivery
3:30 pm #33 Higher Postoperative Radiation Dose Improves The Local Control
Rate For Patients With Positive Surgical Margins And Locally Recurrent
Soft Tissue Sarcoma
Matthew T Ballo, USA
3:45 pm #76 Advantages Of Proton Beams In Treatment Pelvic Sarcomas
Herman Suit, USA
4:00 pm Coffee Break, Poster Viewing
4:00 pm NEW ISSUES IN THE USE OF IMATINIB (Gleevec/Glivec) in GIST and
OTHER SARCOMAS
4:00 pm #29 Phase 2 Trial With Imatinib (glivec, Sti571) In Patients With Gasto-
intestinal Stromal Tumors (gist): Activity Results After 1Year. A Study Of
The Eortc Soft Tissue And Bone Sarcoma Group (stbsg)
Jaap Verweij,The Netherlands
CTOS abstracts S61
4:15 pm #25 (Young Investigator Award Winner) Molecular Targeting Of
PDGF-R By Imatinib Mesylate In Dermato brosarcoma Protuberans
Scott M Schuetze, USA
4:30 pm #57 Gleevec Therapy In C-kit Negative Soft Tissue Sarcomas: A
Molecular Rationale
Dafydd Gareth Thomas, USA
4:45 pm Discussion
5:15 pm Members' Business Meeting
7:00 pm CTOS Reception & Dinner Banquet
Saturday, November 2
6:30 am Registration, Continental Breakfast
7:30 am NEW DATA IN SARCOMA SURGERY
7:30 am #40 Approach To Prosthetic Reconstruction In Sternal Sarcoma
Michele Rocca, Italy
7:45 am #42 Autobiological Reconstruction With Preserved Joint Function After
Surgical Treatment Of Bone Sarcomas In Children
Michele Rocca, Sweden
8:00 am #74 Endoprosthetic Replacement Of Distal Humerus After Tumor
Resection
Fabrice Fiorenza, France
8:15 am #26 Survivorship Analysis Of 141 Modular Metallic Endoprostheses
Mark C. Gebhardt, USA
8:30 am #59 Low Complication Rate With Limb-sparing Resection And
Endoprosthetic Reconstruction: Survival Analysis Of 251 Patients And
Analysis Of 20-year Experience
Felasfa M Wodajo, USA
8:45 am #77 Is There A Case For A Randomized Clinical Trial For Treatment Of
Giant Cell Tumours?
Robert Grimer, UK
9:00 am NINA AXELRAD KEYNOTE LECTURE (supported by the Nina Axelrad Fund)
Dr. Herman Suit:
Perspectives on the Use and Future of Radiation Therapy for Sarcomas
10:00 am Coffee Break, Poster Viewing
10:30 am THE INTERFACE BETWEEN GENETICS AND SARCOMAS:
MINI-SYMPOSIUM ON NF1, Neuro bromatosis, and Malignant Peripheral
Nerve Sheath Tumors
12:30 pm Lunch, Poster Viewing
1:30 pm COLLABORATION AND INNOVATION -
Support and Stimulation of Sarcoma Research
- The view from the US NCI
- The view from the EORTC, Scandinavian, Italian, French, and other Sarcoma
Groups
2:30 pm THE VIEW FROM SARCOMA PATIENT AND FAMILY ADVOCACY GROUPS
GIST - The Life Raft Group
Sarcoma Foundation of America
Sarcoma Alliance
3:30 pm Coffee Break, Poster Viewing
4:00 pm MICRO-SYMPOSIUM: Clinical Trial Statistical Issues for Sarcomas
5:00 pm Adjourn

				

